# Supplementary material for: Causal Relationship between Aging and Anorexia Nervosa: A White-Matter-Microstructure-Mediated Mendelian Randomization Analysis
Source: Biomedicines. 2024 Aug 16;12(8):1874. doi: 10.3390/biomedicines12081874 (PMC11351342; doi:10.3390/biomedicines12081874)
Supplement: Supplementary file 1 [file biomedicines-12-01874-s001.zip › Supplementary Figure/Figure S4/Singe SNP plot of MR analysis for TL to ubm-b-1948.pdf]

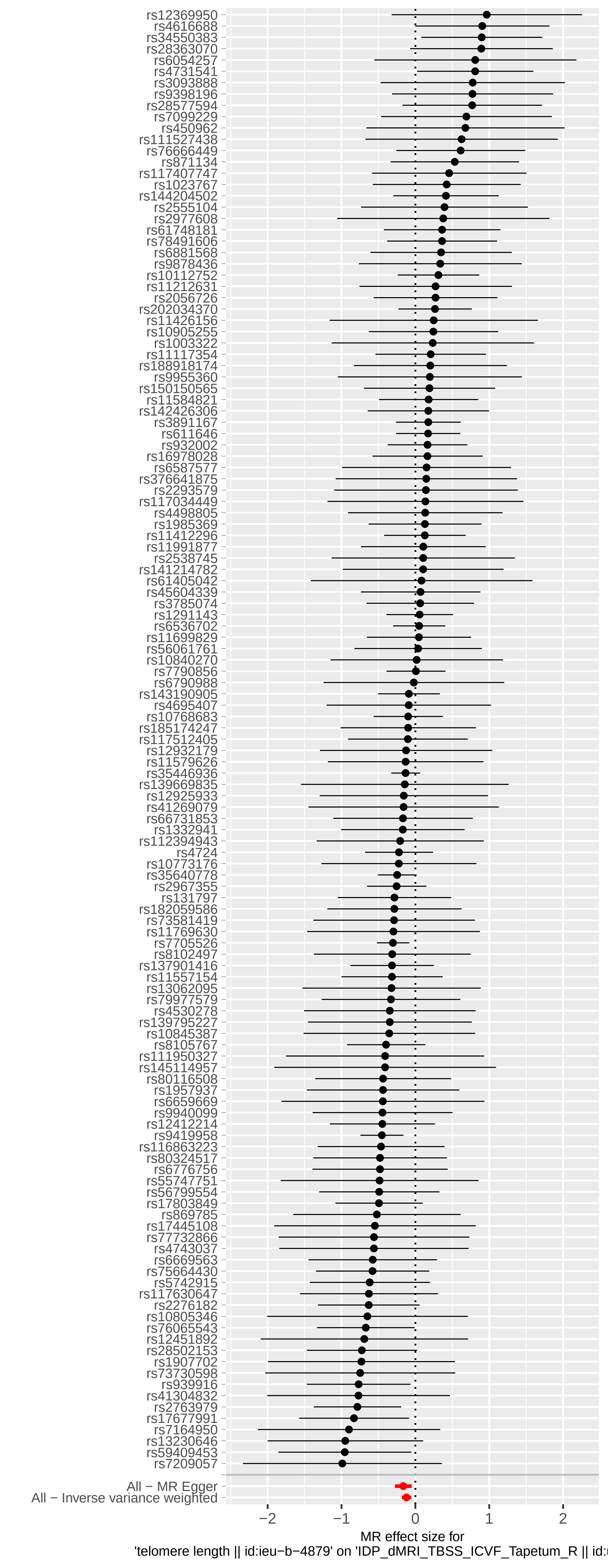
rs12369950
rs4616688
rs34550383
rs28363070
rs6054257
rs4731541
rs3093888
rs9398196
rs28577594
rs7099229
rs450962
rs111527438
rs76666449
rs871134
rs117407747
rs1023767
rs144204502
rs2555104
rs2977608
rs61748181
rs78491606
rs6881568
rs9878436
rs10112752
rs11212631
rs2056726
rs202034370
rs11426156
rs10905255
rs1003322
rs11117354
rs188918174
rs9955360
rs150150565
rs11584821
rs142426306
rs3891167
rs611646
rs932002
rs16978028
rs6587577
rs376641875
rs2293579
rs117034449
rs4498805
rs1985369
rs11412296
rs11991877
rs2538745
rs141214782
rs61405042
rs45604339
rs3785074
rs1291143
rs6536702
rs11699829
rs56061761
rs10840270
rs7790856
rs6790988
rs143190905
rs4695407
rs10768683
rs185174247
rs117512405
rs12932179
rs11579626
rs35446936
rs139669835
rs12925933
rs41269079
rs66731853
rs1332941
rs112394943
rs4724
rs10773176
rs35640778
rs2967355
rs131797
rs182059586
rs73581419
rs11769630
rs7705526
rs8102497
rs137901416
rs11557154
rs13062095
rs79977579
rs4530278
rs139795227
rs10845387
rs8105767
rs111950327
rs145114957
rs80116508
rs1957937
rs6659669
rs9940099
rs12412214
rs9419958
rs116863223
rs80324517
rs6776756
rs55747751
rs56799554
rs17803849
rs869785
rs17445108
rs77732866
rs4743037
rs6669563
rs75664430
rs5742915
rs117630647
rs2276182
rs10805346
rs76065543
rs12451892
rs28502153
rs1907702
rs73730598
rs939916
rs41304832
rs2763979
rs17677991
rs7164950
rs13230646
rs59409453
rs7209057
All − MR Egger
All − Inverse variance weighted
−2
−1
0
1
2
MR effect size for
'telomere length || id:ieu−b−4879' on 'IDP_dMRI_TBSS_ICVF_Tapetum_R || id:
